# Case Report of Atypical Juxtaglomerular Cell Tumor

**DOI:** 10.1155/2018/6407360

**Published:** 2018-10-24

**Authors:** Satoru Munakata, Eisuke Tomiyama, Hitoshi Takayama

**Affiliations:** ^1^Departments of Pathology, Sakai City Hospital Organization, Sakai City Medical Center, Sakai, Japan; ^2^Department of Urology, Sakai City Hospital Organization, Sakai City Medical Center, Sakai, Japan

## Abstract

Juxtaglomerular cell tumor (JGCT) is a rare renal tumor, producing renin and behaving almost in a benign fashion. So far, only three cases have been reported as malignant. We report a rare case with atypical JGCT. A 74-year-old male was referred to our hospital due to hypertension, proteinuria, and hematuria. Abdominal CT revealed a mass measured in 9.7×7.0 cm in the lower portion of the right kidney. Right kidney was removed laparoscopically. Grossly, white to tan tumor with massive hemorrhage and necrosis occupied the lower portion of the right kidney. Microscopically, tumor grew in a solid fashion. Tumor cells were polygonal to ovoid cells with round nuclei and clear to eosinophilic cytoplasm. Mitosis was found in 5 per 10 HPF. Immunohistochemically, tumor cells were stained by vimentin and CD34. Some tumor cells were also positive for renin. Electron micrograph showed near rhomboid crystalline structure in the tumor cells. Because of massive necrosis and mitotic figures, diagnosis of atypical (potentially malignant) JGCT was rendered. Gene mutations for IDH1, PIK3CA, K-ras, N-ras, Braf, and EGFR were not found by MBP-QP system.

## 1. Introduction

Juxtaglomerular cell tumor (JGCT) is a rare renal tumor, first reported by Robertson et al. [1] in 1967, and the name of the tumor was proposed by Kihara et al. [2] in 1968. Since then, about 100 cases have been reported so far [3]. Most of the tumors behave in benign fashion; however, three cases of malignant tumor have been reported [4–6]. We present a rare case of the tumor that is thought to be atypical (potentially malignant).

## 2. Materials and Methods

Immunohistochemical analysis was done by using Bond III system (Leica microsystems, Tokyo, Japan). Primary antibodies were used for cytokeratin (clone AE1/AE3, 1:100, heat, Leica Biosystems Inc., St. Louis, USA), cytokeratin CAM5.2 (clone DC, 1:10, heat, Nichirei Bioscience, Tokyo, Japan), EMA (clone E29, 1:200, Agilent, Santa Clara, CA, USA), PAX8 (polyclonal, 1:100, heat, Proteintech, Rosemont, IL, USA), S-100 (polyclonal, Nichirei), HMB45 (clone HMB45, 1:50, heat, Leica), c-kit (polyclonal, heat, Agilent), CD10 (clone 56C6, 1:100, heat, Leica), MUC-1 (polyclonal, 1:200, heat, Leica), vimentin (clone SRL33, 1:200, heat, Leica), WT-1 (clone 6F-H2, 1:100, heat, Agilent), SMA (polyclonal, 1:300, Agilent), caldesmon (clone h-CD, 1:200, heat, Agilent), CD34 (clone NU-4A1, 1:2, Nichirei), MIB-1 (clone MIB1, 1:100, heat, Agilent), renin (clone EPR20693, 1:200, heat, Abcam, Cambridge, UK), and STAT6 (clone YE361, 1:100, heat, Abcam).

Gene mutations were detected by mutation-biased PCR and quenching probe (MBP-QP) system using i-densy (IS-5320, ARKRAY Inc., Kyoto, Japan) [7]. DNA was extracted from 3 pieces of 5*μ*m paraffin embedded tissue sample by using Maxwell 16® system (Promega Corporation, Tokyo, Japan) after proteinase K treatment (70°C, overnight). Briefly, about 100 bps DNA areas including targeted mutation spots are multiplied by polymerase chain reaction inside i-densy. Then, fluorescent dye conjugated Q-probes that cover targeted mutation spots are hybridized onto the sample DNA. When Q-probes dissociate from sample DNA by heat, the mutations were detected at the QP step by the fluorescence intensity of a TAMRA-conjugated guanine-specific quenching fluorophore probe (QProbe, J-Bio21, Tokyo, Japan). Each probe was designed complementary to each mutation spot. Probes used to analyze mutation include IDH1 (R132X), PIK3CA (exon 9 E542K, exon 9 E545K, exon 20 H1047R), K-ras (codon 12/13, codon 59/61, codon 117, codon 146), N-ras (codon 12/13, codon 59/61), Braf (V600E), and EGFR (exon 18 G719S, G719A, G719C, exon 19 deletion, exon 20 S768I, T790M, exon 21 L858R, L868I).

## 3. Case Presentation

**Figure 1:**
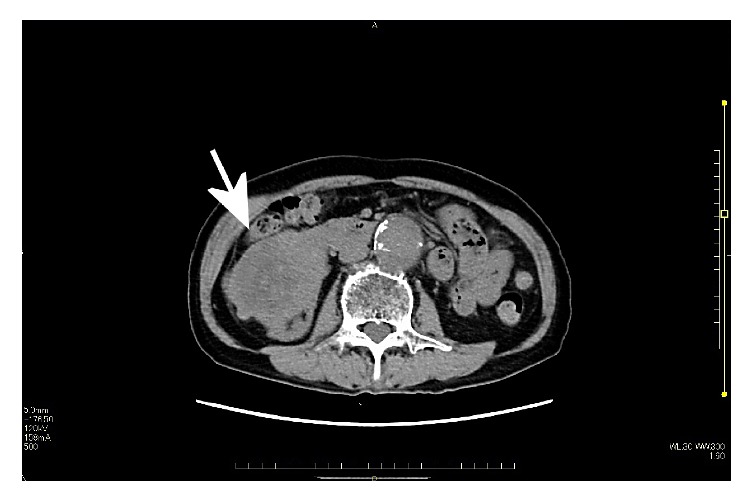
CT image showed right renal mass (arrow).

**Figure 2:**
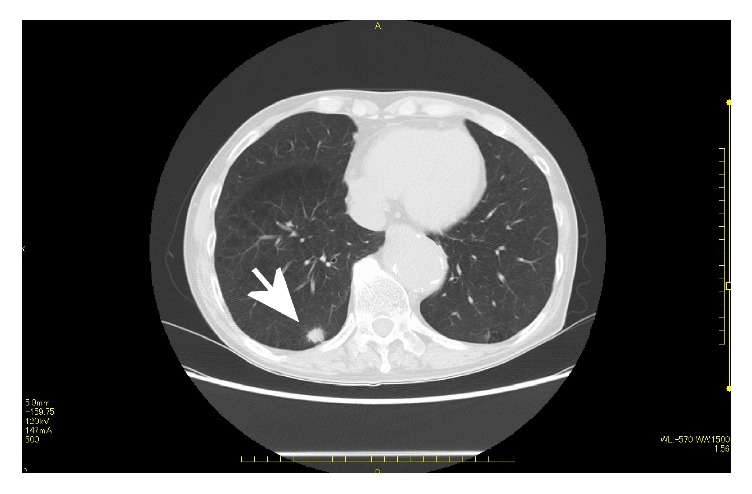
CT image showed right lung mass suspected for metastasis (arrow).

**Figure 3:**
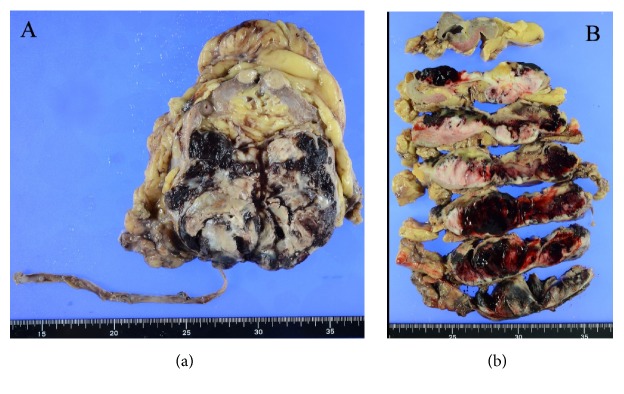
Gross findings of the tumor. 55×94 mm white to tan tumor occupied the lower portion of the right kidney. Marked hemorrhage was found (a, b).

**Figure 4:**
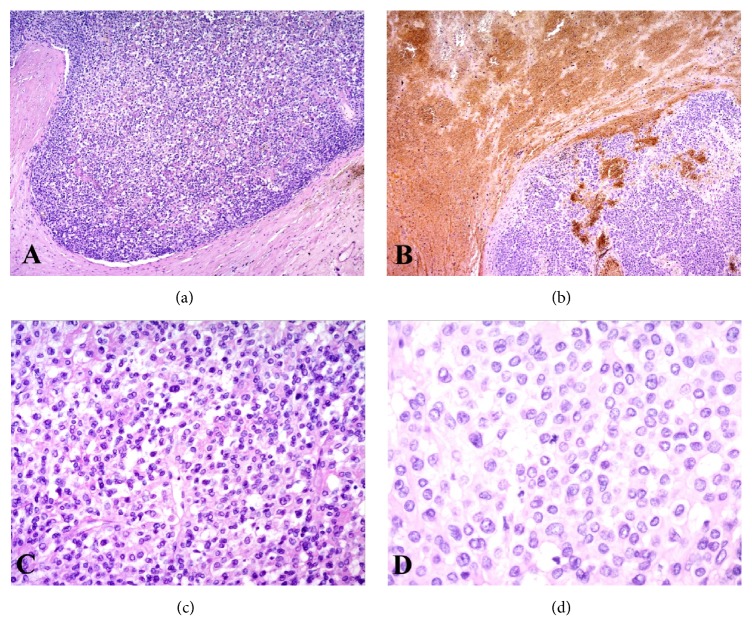
Microscopic findings of the tumor. (a) Tumor cells grow in a solid sheet. (b) Hemorrhage was marked. (c) Tumor cells have relatively bland nuclei and clear to eosinophilic cytoplasm. Cell border is indistinct. (d) Mitosis is observed (hematoxylin & eosin staining, (a, b): x5, (c): x20, (d): x40).

**Figure 5:**
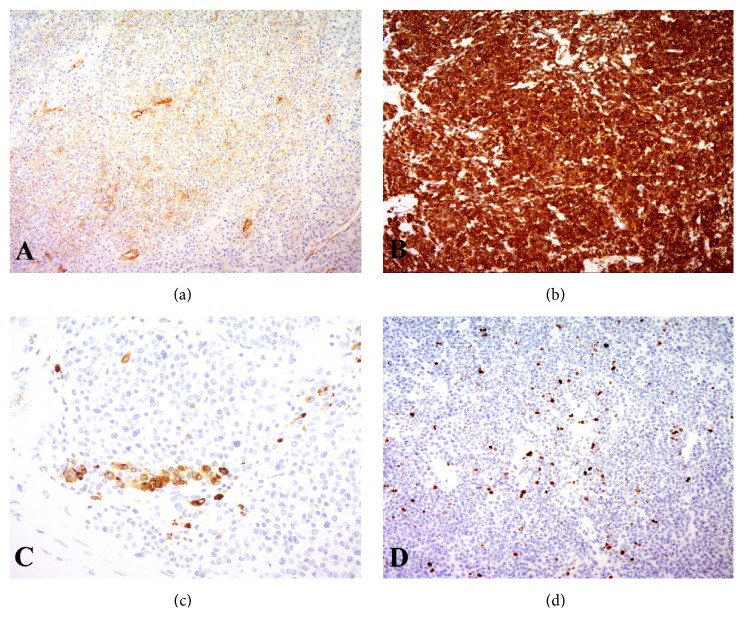
Immunohistochemical analysis. Tumor cells are positive for (a) CD34, (b) SMA, and (c) Renin. (d) MIB1 labeling index was 4% ((a, b, d): x10, (c): x20).

**Figure 6:**
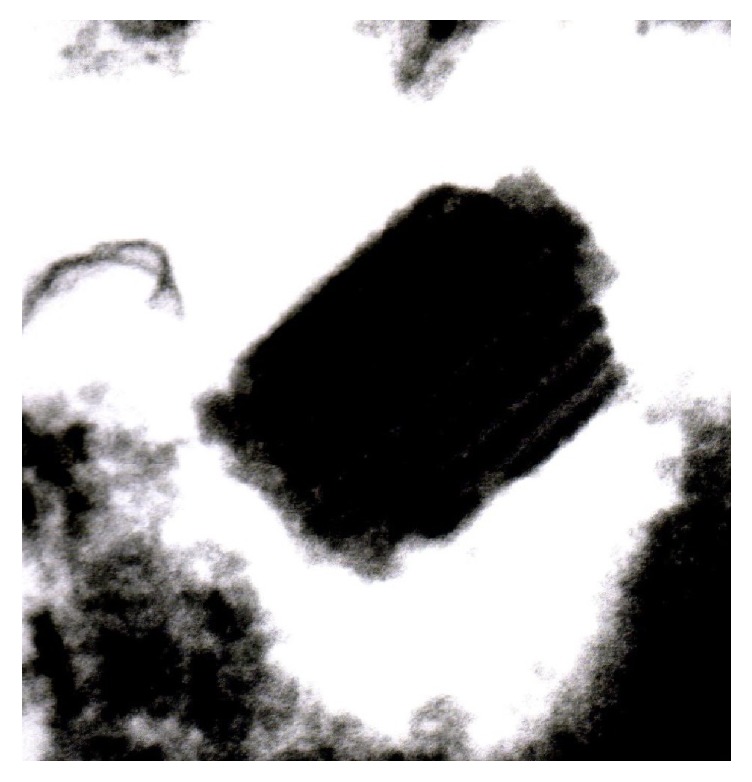
Ultrastructurally, near rhomboid crystalline structures were observed.

A 74-year-old male was referred to our hospital because of hypertension, proteinuria, and hematuria. He was found to have hypertension (BP 146/92 mmHg) and his serum analysis revealed Cr:5.47 mg/dL, UA:11.6 mg/dL, K:6.1 mEq/l. Value of serum tumor markers was high in CEA (7.4 ng/ml), CYFRA (5.7 ng/ml), and proGRP (178.9 pg/ml). His past history was hypertension, and family history was unremarkable. Abdominal CT revealed a mass measured in 9.7×7.0 cm in the lower portion of the right kidney (Figure 1). CT also revealed multiple small nodules in lower lobes of lungs, suspecting metastatic tumors (Figure 2). Laparoscopic right nephrectomy was done for the right renal tumor. Grossly, 55x94 mm white to tan tumor occupied the lower portion of the right kidney (Figure 3). Hemorrhage and necrosis were marked. Microscopically, polygonal to ovoid tumor cells with round nuclei and clear to eosinophilic cytoplasm made solid tumor (Figure 4). Cell border was indistinct. Mitosis was found in 5/10 high power field (Figure 4). Immunohistochemical results are shown in Table 1. CD10, MUC-1, vimentin, WT-1, SMA, caldesmon, and CD34 were positive (Figure 5). Cytokeratin (AE1/AE3), cytokeratin (CAM5.2), EMA, PAX8, S-100, HMB45, c-kit, and STAT6 were negative. Renin was positive in a few tumor cells. MIB1 labeling index was 4% (Figure 5). Ultrastructurally, near rhomboid crystalline structure was observed (Figure 6). Pathological diagnosis was juxtaglomerular cell tumor, malignant. The patient is well 9 months after operation. His serum renin was normal (0.2 ng/ml), 2 months after operation. By follow-up CT, the sizes of multiple lung nodules were stable or diminished, negatively suggesting metastatic tumor, however, not examined histologically. Gene mutations were not found by any probes used in this study.

## 4. Discussion

JGCT is a renin-producing tumor, which causes hypertension, hyperaldosteronism, and hypokalemia. Age prevalence of the tumor is among second and third decades [8]. Symptoms of the patients with JGCT are headaches, retinopathy, double vision, dizziness, nausea, vomiting, polyuria, and proteinuria [7]. Macroscopically, the tumor is yellow to tan and has fibrous capsule. Hemorrhage is frequently associated. Usually, size of the tumor is 3 cm or smaller. Microscopically, round to polygonal tumor cells with clear to eosinophilic cytoplasm and round to oval nuclei grow in solid sheet. Mitotic figures are generally absent. Immunohistochemically, renin reactivity is required, while diffuse vimentin and CD34 staining is observed [8–12]. Immunoreactivity for smooth muscle actin and CD117 varies. Ultrastructurally, rhomboid-shaped renin protogranules are characteristic. In our case, round to polygonal eosinophilic tumor cells grew in a solid sheet. Hemorrhage was also observed. Differential diagnoses include papillary renal cell carcinoma, glomus tumor, hemangiopericytoma, collecting duct carcinoma, urothelial carcinoma, renal epithelioid angiomyolipoma, and Wilms tumor. Immunohistochemically, our tumor was positive for vimentin, CD34, and renin, along with CD10, MUC1, smooth muscle actin, and caldesmon, while it was negative for cytokeratin (AE1/AE3), EMA, CD99, HMB45, and STAT6. Also, near rhomboid-shaped crystalline structure, which could be caused by using formalin-fixed tissue, was observed in the tumor cells in electron micrograph. Negative STAT6 staining excludes solitary fibrous tumor. Renin staining also excludes glomus tumor. By all these findings, JGCT was mostly suspected.

Clinically, hypertension caused by the tumor regresses after resection of the tumor [1, 2]. However, hypertension of our case did not respond well to the resection of the tumor. It is suspected that long standing hypertension might be caused by another reason, i.e., atherosclerosis, because he is a current smoker. Also, this tumor might be nonfunctional as Sakata et al. [13] reported. Indeed, renin-producing cells are relatively few in our tumor by immunohistochemical analysis.

Most of JGCTs behave in a benign fashion. However, three malignant cases have been reported [4–6]. These cases were summarized in Table 2. Except for an 8-year-old boy with local recurrence [6], most of the cases are of older age [4, 5]. Tumor sizes are 8 cm or larger in all of the cases. As Beaudoin stated, potential malignancy of JGCT is suspected if the tumor has histological vascular invasion, the tumor is of a large size, and/or patient's age is relatively advanced [5]. In our case, old age and large size along with necrosis and moderate number of mitotic figures of the tumor suggest malignant potential.

Genetic abnormalities are reported for JGCT. Kuroda et al. [9] reported 6 cases of JGCT and found monosomy of chromosomes 9, 11, and 15 by fluorescence in situ hybridization (FISH). They also observed polysomy of chromosomes 3, 4, 10, 13, 17, and 18 in one case. Oncogenic expression was studied by Wang et al. [12] by immunohistochemistry. BRAF, HER2, and TFE3 genes might not be related in tumorigenesis in JGCTs. Malignant part of the tumor was not examined for genetic abnormalities. So, such a study should be encouraged. In our case, mutations of the genes, including IDH1, PIK3CA, K-ras, N-ras, Braf, and EGFR, were not found by hot spots analysis by MBP-QP system.

In conclusion, we experienced a rare case with atypical JGCT of malignant potential. Close observation of the patient should be recommended.

## Figures and Tables

**Table 1:** Immunohistochemical results.

**Antibody**	**Result**	**Antibody**	**Result**
Cytokeratin (AE1/AE3)	−	WT-1	+

CD10	+	MIB1 labeling index	4%

MUC1	+	LCA	−

Vimentin	+	CD56	−

PAX8	−	CD99	−

PAX2	−	Desmin	−

CAM5.2	−	SMA	+

EMA	−	Synaptophysin	−

CEA	−	Chromogranin A	−

S-100	−	Caldesmon	+

HMB45	−	CD34	+

P53	−	c-kit	−

EGFR	−	Renin	+

PTEN	+	*β*-catenin	+ (cytoplasmic)

STAT6	−	*∙∙∙∙∙*	*∙∙∙∙*

**Table 2:** Reported potentially malignant juxtaglomerular cell tumor.

Age	Sex*∗*	Side	Size (cm)	Reason for malignancy	Reference
52	M	Right	15	Lung metastasis in 6 years	Duan X et al. [4]

51	F	Right	9.8x7.3x8.5	Vascular invasion	Beaudoin J et al. [5]

8	M	Left	8x8	Local recurrence in one year	Shera AH et al.[6]

74	M	Right	5.5x9.4	Necrosis, Mitosis.	Present case
				Lung metastasis?	

*∗*M: male, F: female.
